# Serum microRNA Profiles Reflect Differentiation Status and Age in Early Gastric Cancer

**DOI:** 10.3390/biom16060869

**Published:** 2026-06-13

**Authors:** Marwa Shekfeh, Mariam M. Konaté, Hari Sankaran, Ming-Chung Li, Yingdong Zhao

**Affiliations:** Biometric Research Program, Division of Cancer Treatment and Diagnosis, National Cancer Institute, National Institutes of Health, Rockville, MD 20850, USA

**Keywords:** gastric cancer, miRNA, liquid biopsy, classification, Lasso regression, differentiation status, AYA

## Abstract

Background: Age at diagnosis and histologic differentiation are clinically relevant in early gastric cancer (GC), as poorly differentiated tumors and those diagnosed in younger patients often demonstrate more aggressive characteristics. Serum microRNAs (miRNAs) may provide insights into the molecular basis of these features. Methods: We compared expression profiles between undifferentiated and differentiated early GC cases to identify differentially expressed miRNAs (DEmiRNAs) and associated enriched pathways. Using Lasso regression, we developed and cross-validated a histologic differentiation classifier based on miRNA profiles from 1399 early GC serum samples. Finally, cancer-specific miRNA differences between adolescent and young adult (AYA) and non-AYA patients were evaluated using samples from cancer cases and normal controls. Results: We identified 75 differentiation-associated DEmiRNAs targeting genes enriched in cancer hallmark pathways such as TP53 and PI3K/AKT/mTOR signaling. In the validation set, the combined Lasso model predicted differentiation status with a sensitivity of 69.2%, specificity of 75.3%, positive predictive value (PPV) of 66.9%, negative predictive value (NPV) of 77.2%, an overall accuracy of 73.1%, and an area under the curve (AUC) of 79.7%. Comparison of AYA and non-AYA groups identified 52 cancer-specific and age-related miRNAs. Notably, three components of a previously reported four-miRNA GC diagnostic signature were significantly associated with age. Conclusions: Age-related variation in miRNA expression suggests that patient age may influence the performance of the existing four-miRNA diagnostic signature in early GC. Overall, our findings demonstrate the utility of miRNA profiling for predicting differentiation status in early GC and reveal age-associated variation in cancer-specific miRNAs.

## 1. Introduction

Although gastric cancer (GC) rates are decreasing worldwide, there is a notable increase in incidence within certain age and demographic subgroups, and outcomes remain suboptimal compared to other cancer types [1]. The histologic and molecular landscape of early GC has been studied by several groups [2,3]. The widely used Lauren classification recognizes two subtypes: intestinal and diffuse gastric adenocarcinomas [2], and more recent investigations have characterized additional phenotypes based on molecular and clinical profiles [2]. Though the stepwise progression to intestinal-type gastric adenocarcinoma has been well described, the changes in microRNA (miRNA) expression associated with histologic differentiation (well differentiated vs. poorly or undifferentiated) remain unclear. Poorly differentiated tumors tend to be more aggressive, associated with a worse outcome, and have greater metastatic potential; therefore, a complete resection via endoscopic resection may be unfeasible [4]. Tumor differentiation status can also be a critical factor in clinical trials because certain drugs may be more effective against tumors with specific differentiation characteristics [5].

In clinical practice, an upper gastrointestinal (GI) endoscopy allows physicians to visually examine the stomach lining for abnormalities, such as tumors or lesions. During this procedure, if suspicious areas are identified, biopsies are taken for routine histopathology, which also determines the differentiation status (https://www.cancer.gov/types/stomach/screening, accessed on 6 May 2026). Although the risk of upper GI endoscopic biopsy is minimal, liquid biopsies may be particularly beneficial in the early stages of diagnosis, where the disease is harder to detect even by qualified endoscopists [4,6], or for older patients who have a higher risk associated with the procedure and anesthesia. Furthermore, individuals younger than 40 years old do not routinely undergo screening for GC, making their diagnosis difficult [7]. However, early-onset cases now encompass 5–10% of all GC cases [1,8,9]. Current clinical practice typically involves determining the differentiation status of early GC after diagnosis using methods such as biopsy or gastroscopy. To offer a less invasive and thus more widely accessible alternative, we aimed to develop a model that can help predict differentiation status from serum miRNAs, serving as a supportive tool for clinicians to understand the nature of the tumor.

Circulating miRNAs constitute an attractive option for minimally invasive biomarker testing, due to their functional properties as post-transcriptional regulators and structural features that make them a robust measure, including minimal degradation in biological fluids due to their small size [10,11]. Therefore, the analysis of serum miRNA expression may provide useful information to assist in treatment decisions. In the future, miRNAs may become promising markers or new therapeutic targets for drug response prediction and control [12,13,14].

Given the established association between histologic differentiation and clinical outcomes in GC, we aimed to develop a miRNA signature capable of predicting the differentiation status of early GC. To achieve this, we leveraged publicly available data generated through a comprehensive national project in Japan that profiled circulating miRNAs across 13 human cancer types, including GC. Moreover, because differentiation reflects underlying tumor biology and early-onset GC disproportionately exhibits an undifferentiated phenotype, investigating the relationship between miRNA expression, differentiation status, and age at diagnosis may uncover age-specific regulatory miRNAs associated with an aggressive tumor phenotype. Such miRNAs could provide mechanistic insight into tumor biology and serve as clinically useful biomarkers to inform risk stratification and management of GC.

## 2. Methods

### 2.1. Data Acquisition

The data used in the present study were accessed and downloaded from the National Center for Biotechnology Information Gene Expression Omnibus (GEO) database under the accession number GSE164174 [15]. The authors collected serum samples from 1417 early GC patients between 2008 and 2012 to find a marker for detecting cancer at an early stage [15]. A total of 2565 miRNAs were examined and used in our study. The miRNA signal values were normalized to the ratio of the average signal value of three internal control housekeeping miRNAs (*miR-149-3p*, *miR-2861*, and *miR-4463*).

For the histology association study, we used expression and phenotypic data from cancer patients; we omitted any control samples (i.e., non-cancer) and samples that were labeled as “special” (i.e., neither categorized as well-differentiated nor poorly differentiated). This resulted in 1399 remaining samples, including 811 annotated as “differentiated” and 588 as “undifferentiated.” Table 1 summarizes the relevant demographic characteristics of the patients included in this study, such as sex and age group. The age distribution of GC samples, stratified by binary differentiation status, is illustrated in Figure S1. For the age association study, we incorporated 487 normal serum miRNA profiles obtained from the same data source, i.e., the National Cancer Center BioBank (Tokyo, Japan), as controls.

### 2.2. Intensity Filter

To identify robust and biologically relevant miRNAs, we selected those with a normalized signal value exceeding 64 in more than 50% of the samples, as described in the original study [15]. From the pool of 2565 miRNAs examined, 416 passed this threshold and were selected for further analysis.

### 2.3. Class Comparison of Differentiation Status

Differential expression analysis was performed using the Linear Models for Microarray and RNA-Seq Data (Limma) (v3.64.3) R package, which identifies differentially expressed miRNAs (DEmiRNAs) using linear models. We compared differentiation status and adjusted for two clinical covariates: age and sex. MiRNAs with false discovery rate (FDR) adjusted *p*-value < 0.05 were considered differentially expressed. The FDR-adjusted *p*-values were calculated using the Benjamini–Hochberg method. The differentiated group served as reference, such that miRNAs with log2 fold-change (log_2_FC) < 0 were considered downregulated in the undifferentiated group, while those with log_2_FC > 0 were considered upregulated.

### 2.4. miRNA Enrichment Analysis

We used the RBiomirGS (v0.2.22) [16] R package to conduct the miRNA enrichment analysis. Fold changes (FC) and *p*-values were used to calculate the miRNA score, *S_mirna_*. The *S_mrna_* of the corresponding experimentally validated target mRNA was then calculated as the sign-reversed summation of the *S_mirna_* of all the upstream miRNAs. The combination of *p*-values and the sign of log_2_FC was used to assess both the statistical significance and the direction (upregulation or downregulation in the undifferentiated group) of changes in a more comprehensive manner.

The gene set database file utilized in this study was obtained from the Molecular Signatures Database (MSigDB) website [17,18]. We incorporated 50 Cancer Hallmark gene sets from the Hallmark Gene Set Collection (Hs v6, MSigDB, Broad Institute, Cambridge, MA, USA) and the experimentally validated target mRNAs curated in RBiomirGS to investigate potential associations between these biological processes and miRNA regulation.

In the RBiomirGS output, a positive coefficient for gene set enrichment indicates a potential decrease in miRNA-mediated inhibition of the target mRNA, leading to upregulation of the target genes in the undifferentiated group. Conversely, a negative gene set enrichment coefficient suggests increased miRNA-mediated inhibition of the target mRNA and downregulation of the target genes in the undifferentiated group [16].

### 2.5. Building and Evaluating the Differentiation Status Classifier

We developed three models for predicting sample differentiation status: one using only age and sex, another based only on filtered miRNA expression, and a third integrating both sets of data. This enabled us to compare the predictive capacity of clinical covariates with that of gene expression, and subsequently evaluate whether the expression data provide more accurate predictions than those provided by standard clinical covariates such as age and sex.

To construct the differentiation status prediction model, we employed Lasso (least absolute shrinkage and selection operator) regression [19] with the logistic model implemented within the glmnet R package (v4.1.10). In addition to the traditional 80/20 train-test split, we adopted a more robust internal five-fold cross-validation strategy. This involved partitioning the training set into five folds, iteratively training the model on four folds and evaluating its performance on the remaining fold, before applying the model to the validation set. To select the optimal regularization parameter, lambda, within each training set, we utilized the cv.glmnet function from the glmnet package. This function employs an internal cross-validation approach within each training set to minimize the prediction error, as illustrated in Figure S2.

**Figure 1:**
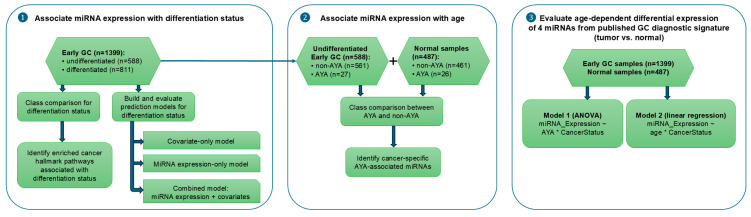
Workflow of the project. The asterisk (*) in the model formulae denotes an interaction term.

We assessed the model performance using a comprehensive set of metrics, including sensitivity, specificity, positive predictive value (PPV), negative predictive value (NPV), accuracy, and the area under the receiver operating characteristic curve (AUROC). All analyses were conducted using R version 3.6.1. Figure 1 provides a graphic overview of the project’s analytical workflow, outlining the key steps involved in model development and evaluation.

### 2.6. Exploratory Analysis of Cancer-Associated miRNAs by Age Group

We performed a class comparison analysis using Limma to evaluate differential miRNA expression between adolescent and young adult (AYA; <40 years) and non-AYA (≥40 years) patients with early GC. MiRNAs with a Benjamini–Hochberg FDR-adjusted *p*-value < 0.05 were considered statistically significant. Differential expression was further defined by an absolute log_2_FC threshold of 0.25, with miRNAs classified as upregulated (log_2_FC > 0.25) or downregulated (log_2_FC < −0.25) in the AYA group.

To account for age-related, non-cancer-specific miRNA variation, a parallel differential expression analysis comparing AYA and non-AYA individuals was conducted in the normal (non-cancer) samples. MiRNAs identified as differentially expressed in both cancer and normal comparisons were excluded. Only those miRNAs uniquely differentially expressed between AYA and non-AYA groups in cancer samples were retained for downstream analysis, thereby enriching for cancer-associated, age-related signals.

Furthermore, to evaluate whether the differential expression of the four miRNAs included in the GC diagnostic model developed by Abe et al. (*miR-4257*, *miR-6785-5p*, *miR-187-5p*, and *miR-5739*) varied by age, we fit the following two models:

Model 1 (ANOVA): miR_Expression ~ AYA * cancer status

Model 2 (linear regression): miR_Expression ~ Age * cancer status

The interaction terms were used to assess whether tumor–normal expression differences varied by binary age group (AYA, non-AYA) or continuous age. Cancer status is an indicator of whether the sample was obtained from a patient with GC or a normal control. Using both models allows assessment of age effects under both a categorical (AYA vs. non-AYA) and a continuous representation of age, providing complementary perspectives and improving robustness of the findings.

## 3. Results

### 3.1. Identification of miRNAs Associated with Differentiation Status in Early GC

**Figure 2:**
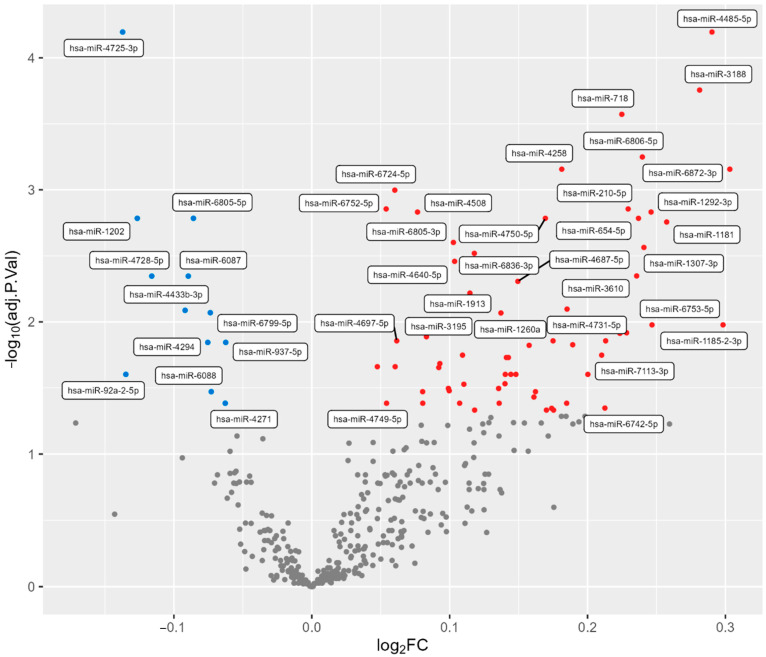
Volcano plot of DEmiRNAs between undifferentiated and differentiated samples out of 416 filtered miRNAs. The red dots represent the 63 upregulated miRNAs, and the blue dots represent the 12 downregulated miRNAs. Non-significant miRNAs are shown in gray. The threshold for defining a miRNA as differentially expressed was set at an adjusted *p* < 0.05.

DEmiRNAs were identified with FDR-adjusted *p* < 0.05. Among the 416 miRNAs analyzed using Limma, 75 miRNAs were significantly differentially expressed between undifferentiated and differentiated GC samples (Table S1). Of these, 63 miRNAs were upregulated, and 12 were downregulated, as shown in Figure 2. The top five upregulated miRNAs in undifferentiated GC, ranked by *p*-value and log_2_FC, are *hsa-miR-4485-5p*, *hsa-miR-3188*, *hsa-miR-718*, *hsa-miR-6806-5p*, and *hsa-miR-4258*. The top five downregulated miRNAs are *hsa-miR-4725-3p*, *hsa-miR-1202*, *hsa-miR-6805-5p*, *hsa-miR-4728-5p*, and *hsa-miR-6087*.

Significant DEmiRNAs were mapped to previously curated functional miRNA sets collected in TAM 2.0 [20]. We matched our DEmiRNAs to 21 previously annotated miRNA functional sets, including apoptosis, epithelial-to-mesenchymal transition (EMT), cell death, and onco-miRNAs (Table S2). For instance, *hsa-miR-92a,* with suggested diagnostic and prognostic value in GC [21,22] and which came up as downregulated in serum from patients with undifferentiated early GC belongs to multiple miRNA sets, including aging, apoptosis, cell cycle, cell death, and proliferation, and onco-miRNA. Another onco-miRNA, *hsa-miR-210*, which was upregulated in undifferentiated early GC in our analysis, is also involved in multiple cancer-related processes such as the cell cycle and EMT. Finally, the following DEmiRNAs in our analysis are categorized under gastric carcinoma in TAM 2.0, which supports our results and confirms that these miRNAs participate in the biology of GC: *hsa-miR-210*; *hsa-miR-423*; *hsa-miR-551b*; *hsa-miR-128-1*; *hsa-miR-4697*; *hsa-miR-92a-2*; *hsa-miR-128-1*; and *hsa-miR-92b*.

### 3.2. Exploration of Enriched Cancer Pathways Associated with Differentiation Status

**Figure 3:**
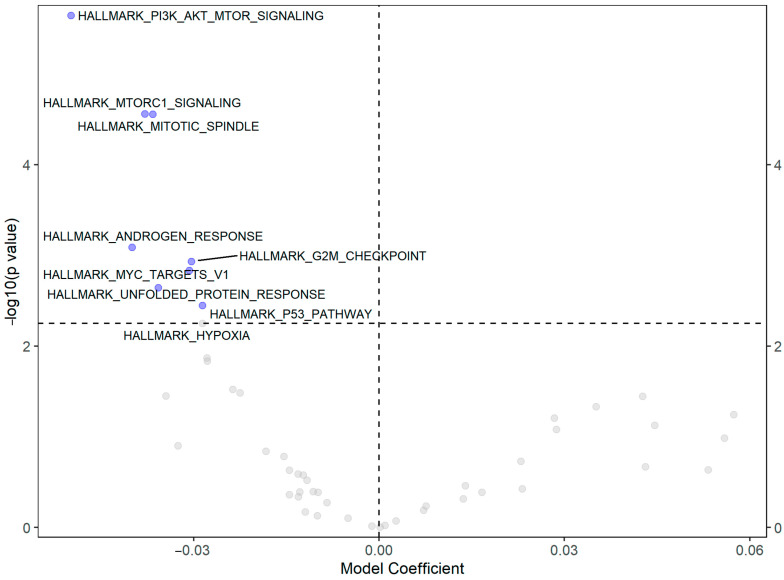
Volcano plot of enriched Cancer Hallmark pathways between undifferentiated and differentiated early GC samples tested with RBiomirGS. Significantly enriched gene sets with an adjusted *p* < 0.05 are represented as blue circles, whereas non-significant gene sets are shown in gray. The horizontal dashed line indicates the FDR-adjusted *p* < 0.05 threshold, while the vertical dashed line indicates the sign of the model coefficient.

Several studies have implicated miRNA dysregulation in the initiation and progression of various cancers [23]. To uncover pathways associated with differentiation status in early GC, we conducted pathway enrichment analysis of the DEmiRNAs using RBiomirGS [16]. Table S3 contains the input list of miRNAs and differential expression statistics for RBiomirGS, and a volcano plot of the resulting significantly enriched cancer hallmark pathways appears in Figure 3. Cancer hallmark pathways with an FDR-adjusted *p* < 0.05 and their respective coefficient scores are listed in Table S4. As per the original paper describing the RBiomirGS package, a gene set with a positive model coefficient might be under more miRNA-dependent inhibition in the differentiated group and less inhibition in the undifferentiated group, and the reverse applies to negative model coefficients.

Nine pathways exhibit negative model coefficient values (Figure 3): PI3K–AKT–mTOR signaling, mTORC1 signaling, mitotic spindle, androgen response, G2M checkpoint, MYC targets, unfolded protein response, TP53, and hypoxia pathways. These pathways undergo increased miRNA inhibition, corresponding to downregulation of target genes in the undifferentiated group.

### 3.3. Development of a Prediction Model for Differentiation Status

We initially built a classifier that considered only the demographic parameters (age + sex) as covariates. The model’s overall accuracy on the validation set was 66.3%. Although sensitivity was low at 59.8%, the specificity of the model was 71.0%. The PPV was 59.8%, and the NPV was 71.0%. The low sensitivity of the model made it very weak in recognizing differentiated samples, with correct predictions in <60% of cases, although the overall accuracy was better than random. These results are summarized in Supplementary Table S5.

Then, to assess whether expression data alone could accurately predict differentiation status, we built a predictor model using Lasso regression, considering only miRNA expression data. This model achieved a classification accuracy of 72.4% in the validation set (Table S6). This model’s sensitivity reached 74.4% while specificity was calculated as 71%. The PPV and NPV were calculated as 64.9% and 79.3%, respectively.

**Figure 4:**
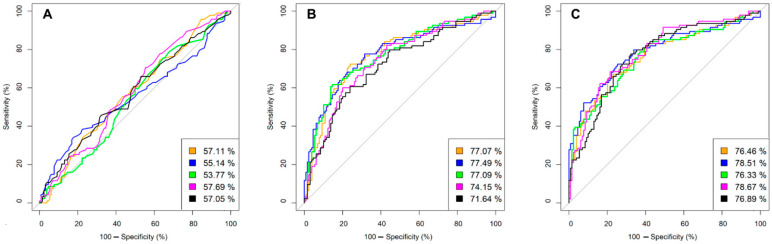
Cross-validation ROC curves discriminating differentiated samples from undifferentiated samples. Different colors represent the ROC curves for each fold of cross-validation while training the model, and the gray line represents a random classifier. (**A**) Covariate-only model, (**B**) expression-only model, and (**C**) combined data model.

Finally, we built a predictor model based on both demographic characteristics and miRNA expression data (Table S7). In this combined model, sensitivity was 69.2%, and specificity was 75.3%. The accuracy of the model was 73.1%. The accuracy of the combined model was only marginally better than that of the expression-only model (Table S6). Figure 4 illustrates the performance of the three models during training and compares the corresponding AUC values. In the combined model, AUC varied between 76.3% and 78.7% (Figure 4C), suggesting that the model’s overall performance was sufficient to distinguish between differentiated and undifferentiated GC serum samples. Table 2 summarizes the performance metrics of the three models. The respective detailed tables are also supplied in the Supplementary Material of this paper (Tables S5–S7). Additionally, the 39 miRNAs selected by Lasso for the combined model are featured in Figure S3 and listed in Table S8.

### 3.4. Exploratory Analyses Using AYA Group and Age

As part of an exploratory analysis aimed at characterizing age-associated molecular differences within a specific histologic subgroup of GC, patients were stratified into AYA (age < 40, *n* = 28) and non-AYA (age ≥ 40, *n* = 1371) groups to evaluate differential expression of miRNA in early GC cases. As shown in Table 1, the majority of the AYA patients (27 out of 28) had GC tumors classified as having undifferentiated histology; therefore, undifferentiated AYAs (*n* = 27) were compared to undifferentiated non-AYAs (*n* = 561).

Among 416 miRNAs, 110 were significantly differentially expressed between AYA and non-AYA groups in cancer samples at an FDR-adjusted *p*-value < 0.05. In parallel, 105 miRNAs were identified as significantly differentially expressed between AYA and non-AYA groups in normal samples. Applying an additional threshold of |log_2_FC| > 0.25, 84 miRNAs remained differentially expressed in cancer samples and 91 in normal samples. After excluding miRNAs that were also differentially expressed in normal samples, 52 miRNAs were identified as cancer-specific, age-associated miRNAs. Downregulated miRNAs in AYA compared to non-AYA include *hsa-miR-5739*, *hsa-miR-1185-2-3p*, *hsa-miR-6802-5p*, *hsa-miR-1202*, and *hsa-miR-3162-5p* (Table S9), several of which have been identified as having tumor-suppressing properties [24,25,26,27]. Upregulated miRNAs in the serum of AYA patients include *hsa-miR-6784-5p*, *hsa-miR-762*, *hsa-miR-3621*, *hsa-miR-3940-5p*, and *hsa-miR-4446-3p* (Table S9).

**Figure 5:**
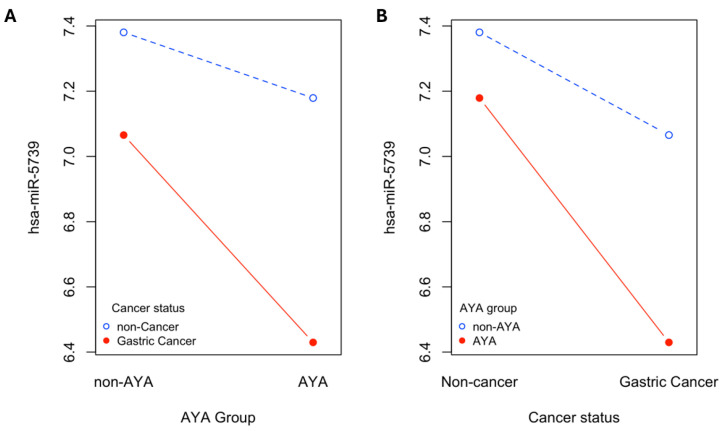
Interaction between cancer status and AYA group on *hsa-miR-5739* expression. Interaction plots showing mean expression levels of *hsa-miR-5739* stratified by cancer status (non-cancer vs. cancer) and AYA group (non-AYA vs. AYA). (**A**) AYA group is shown on the x-axis, with separate lines representing cancer status. (**B**) Cancer status is shown on the x-axis, with separate lines representing AYA group. In both panels, the non-parallel lines indicate a significant interaction between cancer status and AYA group, suggesting that the effect of cancer status on *hsa-miR-5739* expression differs by AYA group.

Abe et al. [15] reported a highly accurate serum miRNA-based model for the detection of early GC. The identification of a four-miRNA panel (*miR-4257*, *miR-6785-5p*, *miR-187-5p*, and *miR-5739*) with excellent diagnostic performance represents an important step toward non-invasive early detection. We evaluated whether the differential expression of the four miRNAs between tumor and normal samples varied by age using two models (Table 3). In the ANOVA model with AYA status, significant interactions between cancer status and AYA were observed for *miR-187-5p* (*p* = 0.0011) and *miR-5739* (*p* = 0.0048), indicating that tumor–normal differences for these miRNAs vary by AYA status. In the linear regression model with age as a continuous variable, significant interactions were identified for *miR-6785-5p* (*p* = 0.0007) and *miR-5739* (*p* = 0.0098), with a borderline interaction for *miR-187-5p* (*p* = 0.0744). No significant interaction was observed for *miR-4257* in either model. Figure 5 demonstrates a significant interaction between cancer status and AYA status for *hsa-miR-5739* expression. These findings suggest that the effect of cancer status (tumor vs. normal) on the expression of several miRNAs in the original diagnostic model varies by age, as captured by AYA status or continuous age.

## 4. Discussion

Differentiation status is an important factor associated with lymph node metastasis and prognosis. It also affects endoscopic resectability (as opposed to more burdensome surgery), as undifferentiated tumors are more difficult to remove via minimally invasive endoscopic resection.

MiRNAs have emerged as important regulators of cellular metabolic pathways, and recent research has demonstrated their role in cancer cell pathophysiology. Consistent with our findings, most of the miRNAs identified in this study have been previously reported to be significantly dysregulated in GC, thus playing important roles in tumor development. MiRNAs in the context of GC may either act as tumor suppressors or proto-onco-miRNAs [4]. A recent paper by Yu et al. reviewed studies linking miRNAs to the regulation of the tumor microenvironment (TME) [28]. The authors note that some miRNAs are known to modulate immune cells, drive metastasis and chemoresistance, and reprogram cancer-associated fibroblasts (CAFs). Notably, viral infections (e.g., *Helicobacter pylori* and Epstein–Barr virus) can reshape the immune TME via miRNA regulation.

The PI3K–AKT–mTOR and mTORC1 pathways are associated with cell growth and survival, and are controlled in part by the TP53 signaling pathway. However, this pathway is frequently altered in cancer, including in GI tumors [29], and is shown in Figure 3 among the significantly downregulated gene sets in undifferentiated GC, probably due to the presence of inactivating mutations. This signaling pathway is also known as a prognostic biomarker, related to tumor recurrence and chemotherapy resistance [30], and relates to the induction of post-transcriptional maturation of different suppressor miRNAs [29].

Our analysis identified DEmiRNAs whose validated target genes are enriched in signaling pathways frequently altered in cancer, including PI3K, mTOR, TP53, and G2M pathways. The miRNA *miR-486-5p* (upregulated, Table S1) was found to be important for cell proliferation, invasion, and migration [31]. This miRNA is a known modulator of GC cell proliferation, migration, and tumor progression [31,32]. Another notable miRNA, *miR-210*, is upregulated in multiple cancers and was even found to be expressed in inflammatory cells of the TME [33]. In our analysis, *miR-210-5p* overexpression was noted in undifferentiated tumors. Interestingly, several groups have demonstrated that *miR-210* overexpression in response to cellular stress due to hypoxia or DNA damage could induce oncogenic autophagy or senescence, leading to the inhibition of the PI3K/AKT/mTOR signaling pathway [34,35]. Wei et al. developed a senescence-associated gene signature in gastric cancer samples from The Cancer Genome Atlas and uncovered a strong association between senescence gene expression profiles and tumor histological grade [36]. Liu et al. found that *miR-210-5p* suppressed AKT/mTOR via *PIK3R5* in osteosarcoma [37]. Moreover, Bi et al. showed that *miR-210* could inhibit PI3K, AKT, and mTOR protein expression in M2 macrophages and induce autophagy [38]. This disruption of the TME, in turn, promoted the proliferation and invasiveness of co-cultured hepatocellular carcinoma cells [38]. Furthermore, *miR-92b,* which is overexpressed in undifferentiated GC, was elevated in response to autophagy-inducing stimuli in breast cancer [39] and was demonstrated to promote chemoresistance in colorectal cancer in vitro and in vivo through regulation of cell cycle and apoptosis by targeting *CDKN1C* [40]. These results are consistent with the predicted inhibition in undifferentiated GC of genes involved in proliferative pathways such as PI3K/AKT/mTOR, G2M, and MYC (Figure 3). Nonetheless, given that the RBiomirGS pathway enrichment analysis was derived from validated target genes of circulating miRNAs rather than matched tumor transcriptomic data, these findings should be interpreted cautiously and warrant further validation in independent molecular datasets.

Classification based on serum samples provides an opportunity for better diagnosis and treatment of GC. Indeed, serum collection, as a less invasive alternative to gastroscopy, would lessen the strain on patients. Here, we report a refined GC classification method based on miRNA associated with distinct clinical outcomes. In this exploratory study, we analyzed a cohort of 1399 tumor samples representing two different histology types of GC. We developed a classifier for GC histologic differentiation based on the serum levels of 39 miRNAs. The classifier demonstrated a sensitivity of 74.4% and a specificity of 71%, with an accuracy of 72.4%. To our knowledge, this is among the best ranges of accuracy achieved in previous studies, given that GC is histologically complex and sometimes transitions from a differentiated to an undifferentiated type within the same tumor (mixed type). Furthermore, the results described in this paper are specific to early GC, where endoscopic detection, the gold standard for diagnosis, lacks accuracy due to the subtle mucosal changes that make the disease harder to visualize [41]. The combined model marginally improved accuracy (73.1% vs. 72.4% for the expression-only data model and 66.3% for the covariate data model). However, sensitivity was lower in the combined data model compared to the miRNA expression model (69.2% vs. 74.4% Table 2).

Other studies have focused on “black box” machine learning models such as convolutional neural networks, which are harder to interpret than the model we present here, and most focus on predicting the presence or recurrence of cancer rather than differentiation status. For instance, Wu et al. used a proprietary artificial intelligence (AI) system, ENDOANGEL, to predict different clinicopathological features of early GC. The performance of AI to predict differentiation status was better than that of endoscopists [42]. The authors noted differentiation status prediction accuracy of 71.4%, sensitivity of 50%, and specificity of 80%. Our approach further improved accuracy (72.4% for the expression-only model and 73.1% for the combined model) and sensitivity (74.3% for the expression-only model and 69.2% for the combined model) compared to those reported for the AI.

GCs occurring in AYA patients tend to be poorly differentiated, larger, and have a greater prevalence in females [43]. In this dataset, all but one of the AYA cases were undifferentiated (Table 1). With the noted trend towards a higher incidence of GC among younger cohorts [1], these results highlight the importance of developing tools to diagnose and characterize undifferentiated GC in these patients. We uncovered substantial age-related variation in miRNA levels even within the same differentiation status, supporting the broader hypothesis that age-associated biological effects may influence serum miRNA profiles. Further, our analyses indicate that the effect of cancer status (tumor vs. normal) on the expression of three miRNAs in the original four-miRNA GC diagnostic model [15] is modified by age, whether modeled as AYA status or as a continuous variable. These findings suggest that age should be considered when applying the four-miRNA GC diagnostic predictor.

One notable limitation of our study is that it was based on a single dataset and on a homogeneous population from a national project in Japan, without an appropriate validation dataset of serum miRNA expression data in early GC. It is also known that there is an intermediate state between differentiated and undifferentiated tumors; therefore, drawing a binary cutoff, as was the case in the dataset used for this study [15] may not be a suitable approach, as prediction accuracy for any partially differentiated tumor may be diminished. A further limitation of this study is the absence of relevant clinical and phenotypic variables in the dataset, such as survival data, gastric cancer subtype, *H. pylori* infection, and smoking status [3], which could not be incorporated into the model during training. These characteristics and risk factors may also influence the levels of circulating miRNAs [1,44] and thus, their inclusion in a classification model could improve the prediction accuracy substantially.

## 5. Conclusions

The current work represents an initial exploratory investigation rather than a definitive study of translational validity. To our knowledge, this study is one of the earliest applications of machine learning to serum miRNA data for classifying the differentiation status of GC samples. We evaluated whether a prediction model for differentiation status could be established from serum miRNA profiles of patients with early GC. Importantly, we observed age-related variation in the expression of the previously reported four-miRNA diagnostic signature for early GC. This finding suggests that patient age may influence the performance of this classifier and should be considered in its clinical application. Future validation using independent multi-center cohorts and alternative platforms will be necessary to confirm the reproducibility and clinical applicability of the identified miRNA signatures.

## Figures and Tables

**Table 1:** Clinical characteristics of the data.

	Differentiated *n* (%)	Undifferentiated *n* (%)	Fisher’s Exact Test *p* Value
Age			<0.0001
AYA (<40)	1 (0%)	27 (5%)	
Non-AYA (≥40)	810 (100%)	561 (95%)	
Sex			<0.0001
Female	172 (21%)	241 (41%)	
Male	639 (79%)	347 (59%)	

**Table 2:** Performance of different prediction models in the validation set. Each row represents one of the three models.

	Sensitivity	Specificity	PPV	NPV	Accuracy	AUC	Number of Predictors in the Full Model
Covariate Data Model	0.5983	0.7099	0.5983	0.7099	0.6631	0.7053	2
miRNA Expression Data Model	0.7436	0.7099	0.6493	0.7931	0.724	0.7849	50
Combined Model	0.6923	0.7531	0.6694	0.7722	0.7308	0.7971	41 *

* 41 predictors include 39 miRNAs and two clinical covariates (age and sex).

**Table 3:** *p*-values for interaction terms between age-related variables and cancer status across four miRNAs. Model 1 (ANOVA) includes the interaction between AYA status and cancer status (AYA * cancer status), while Model 2 (linear regression) includes the interaction between continuous age and gastric cancer status (Age * cancer status).

	Model 1 AYA * Cancer Status	Model 2 Age * Cancer Status
*miR-6785-5p*	0.6053	0.0007
*miR-187-5p*	0.0011	0.0744
*miR-4257*	0.9686	0.3257
*miR-5739*	0.0048	0.0098

The asterisk (*) in the model formulae denotes an interaction term.

## Data Availability

The original dataset by Abe et al. is publicly available on the GEO website under the accession number GSE164174 [15]. The R code will be made available upon request.

## Supplementary Materials

The following supporting information can be downloaded at: https://www.mdpi.com/article/10.3390/biom16060869/s1, Figure S1: Age distribution of GC samples stratified by binary differentiation status; Figure S2: Lasso model analysis; Figure S3: Volcano plot of Lasso selected miRNAs; Table S1: List of DEmiRNAs and the corresponding *p*-values and log2FC values; Table S2: Mapping DEmiRNAs to 20 gene sets annotated by TAM 2.0; Table S3: Input table for RbiomirGS, which includes the DEmiRNA name, FC and *p*-value; Table S4: Output table from RbiomirGS enrichment, which includes cancer hallmark gene sets and the corresponding t-value, *p*-value, and FDR-adjusted *p*-value; Table S5: Covariates-only model performance metrics; Table S6: Expression data-only model performance metrics; Table S7: Combined model performance metrics; Table S8: List of the 39 miRNAs selected by Lasso for the combined prediction model; Table S9: DEmiRNAs in serum samples from AYA vs. non-AYA patients with GC.

## Abbreviations

The following abbreviations are used in this manuscript:

AI	artificial intelligence
ANOVA	analysis of variance
AUC	area under the curve
AUROC	area under the receiver operating characteristic
AYA	adolescent and young adult
CAF	cancer-associated fibroblast
DEmiRNA	differentially expressed miRNA
EGC	early gastric cancer
EMT	epithelial-to-mesenchymal transition
FC	fold-change
FDR	false discovery rate
GC	gastric cancer
GEO	gene expression omnibus
GI	gastrointestinal
Lasso	least absolute shrinkage and selection operator
Limma	linear models for microarray and RNA-seq data
Log_2_FC	log_2_ fold-change
miR	microRNA
miRNA	microRNA
MSigDB	molecular signatures database
NPV	negative predictive value
PPV	positive predictive value
ROC	receiver operating characteristic
TME	tumor microenvironment
