# Supplementary material for: Serum microRNA Profiles Reflect Differentiation Status and Age in Early Gastric Cancer
Source: Biomolecules. 2026 Jun 13;16(6):869. doi: 10.3390/biom16060869 (PMC13296932; doi:10.3390/biom16060869)
Supplement: Supplementary file 1 [file biomolecules-16-00869-s001.zip › Biomolecures-4334600-supplementary.pdf]

Serum microRNA Profiles Reflect Differentiation Status and Age in Early Gastric Cancer

Marwa Shekfeh, Mariam M. Konaté, Hari Sankaran, Ming-Chung Li, Yingdong Zhao *

Biometric Research Program, Division of Cancer Treatment and Diagnosis, National Cancer Institute, National Institutes of Health, USA.

*Corresponding author

Supplementary Figures:

- Figure S1: Age distribution of GC samples stratified by binary differentiation status.
- Figure S2: Lasso model analysis.
- Figure S3: Volcano plot of Lasso selected miRNAs.

Supplementary Tables (in a separate Excel file):

- Table S1: List of DEmiRNAs and the corresponding *p*-values and log2FC values.
- Table S2: Mapping DEmiRNAs to 20 gene sets annotated by TAM 2.0.
- Table S3: Input table for RbiomirGS, which includes the DEmiRNA name, FC and *p*-value.
- Table S4: Output table from RbiomirGS enrichment, which includes cancer hallmark gene sets and the corresponding t-value, *p*-value, and FDR-adjusted *p*-value.
- Table S5: Covariates-only model performance metrics.
- Table S6: Expression data-only model performance metrics.
- Table S7: Combined model performance metrics.
- Table S8: List of the 39 miRNAs selected by Lasso for the combined prediction model.
- Table S9: DEmiRNAs in serum samples from AYA vs. non-AYA patients with GC.

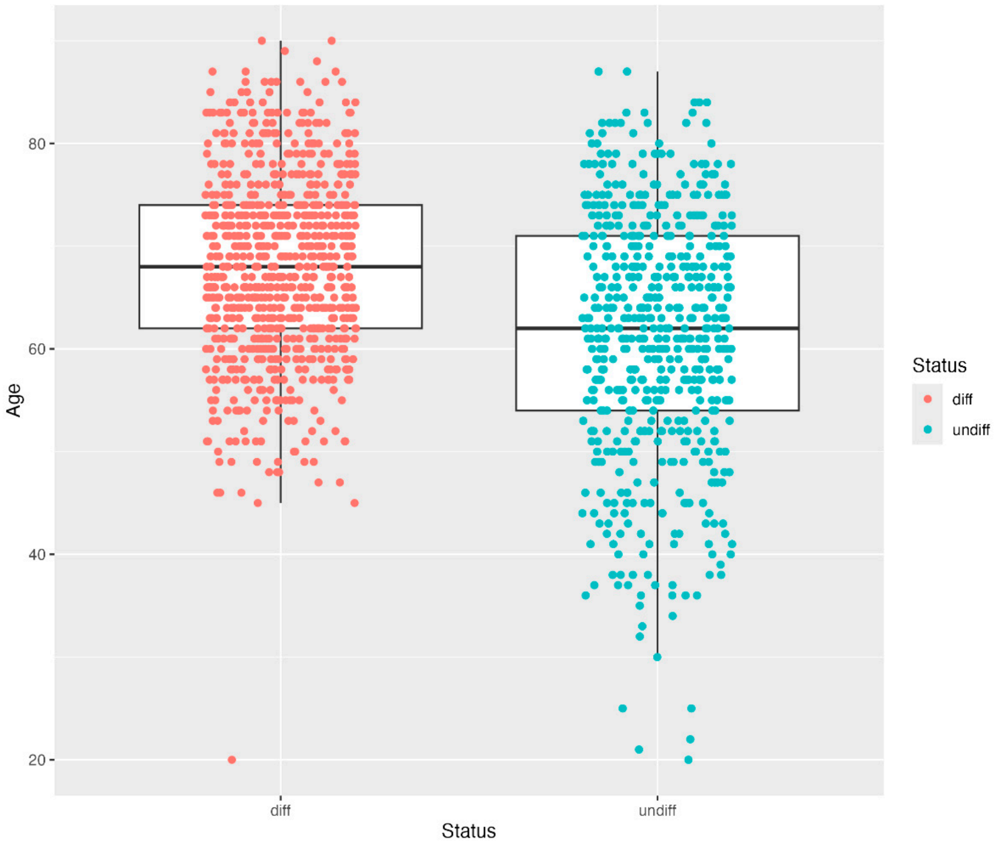

Figure S1. Age distribution of GC samples stratified by binary differentiation. Diff, differentiated; undiff, undifferentiated. Age and differentiation status data was obtained from reference 15.

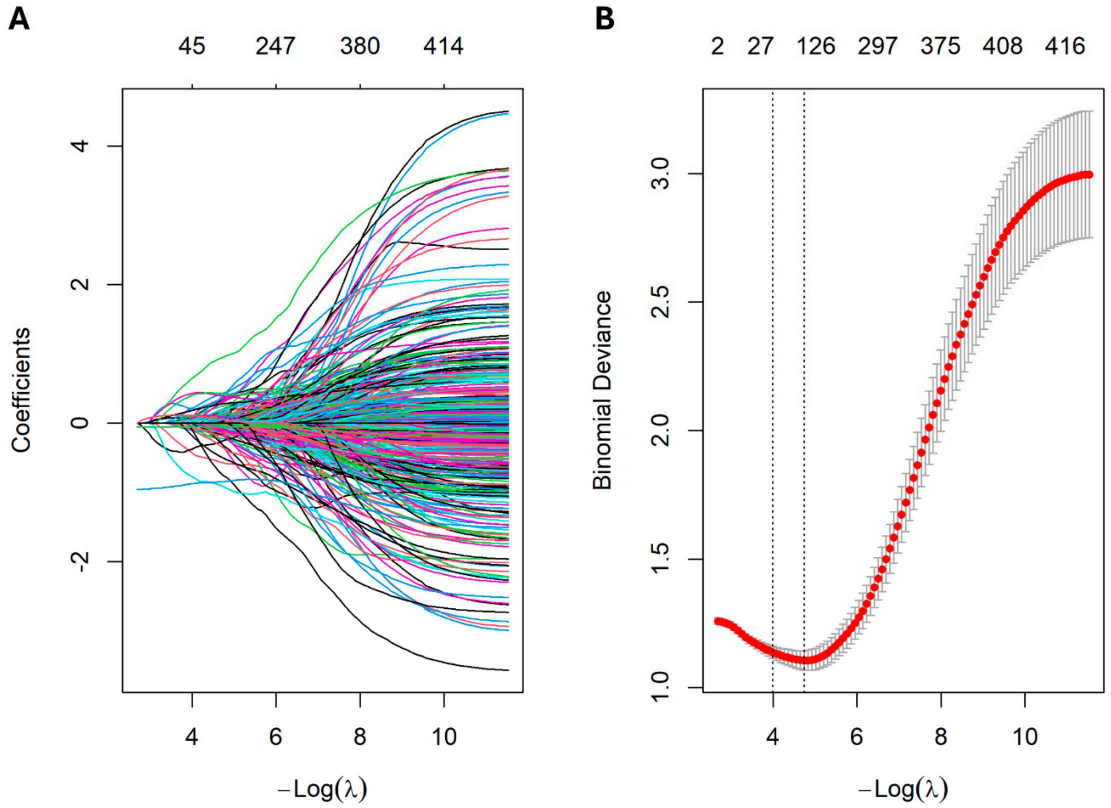

Figure S2. Lasso model analysis. (a) Lasso coefficient profiles for each variable and (b) cross-validation curve with upper and lower standard deviation (grey error bars). The dotted lines along the λ sequence indicate the value of λ that minimizes the mean cross-validated error (lambda.min), and the value of λ that results in the most regularized model such that the cross-validated error is within one standard error of the minimum (lamda.1se), respectively.

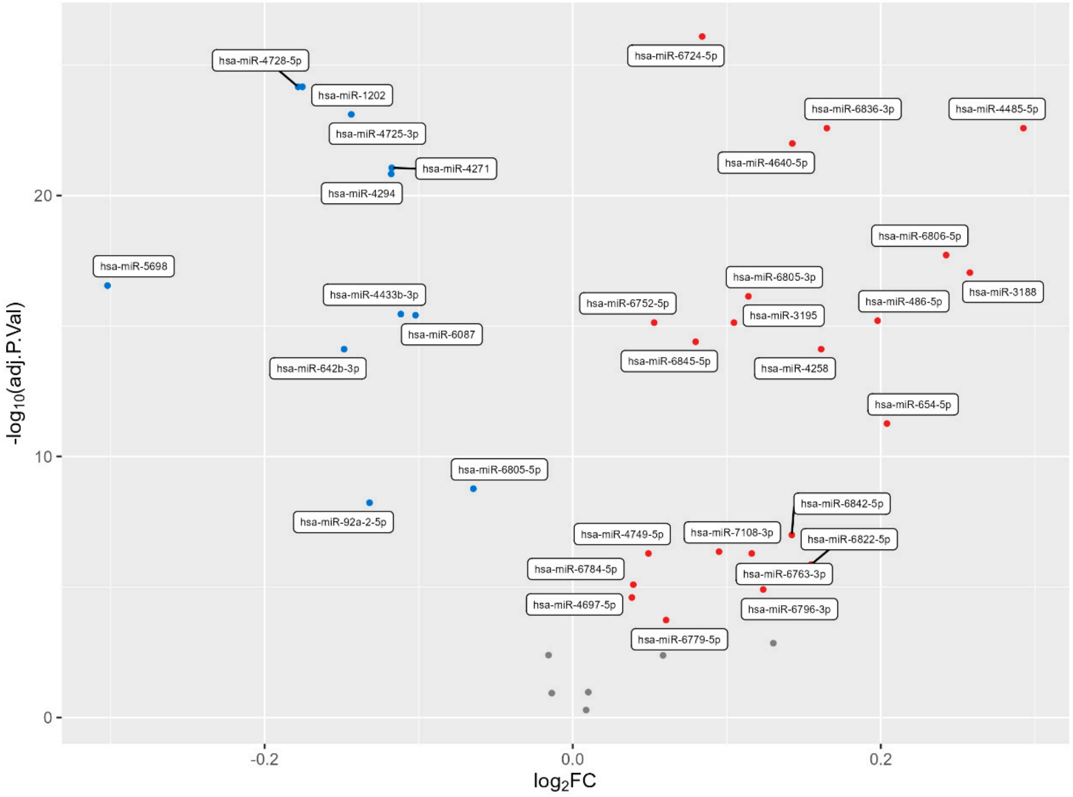

Figure S3. Volcano plot of Lasso selected miRNAs. The red dots represent upregulated miRNAs, and the blue ones represent downregulated miRNAs. The cutoff for considering a miRNA as deregulated is FDR-adjusted $p < 0.05$.
